# Immunological Homeostasis at the Ovine Placenta May Reflect the Degree of Maternal Fetal Interaction

**DOI:** 10.3389/fimmu.2018.03025

**Published:** 2019-01-09

**Authors:** Sean R. Wattegedera, Laura E. Doull, Mariya I. Goncheva, Nicholas M. Wheelhouse, Donna M. Watson, Julian Pearce, Julio Benavides, Javier Palarea-Albaladejo, Colin J. McInnes, Keith Ballingall, Gary Entrican

**Affiliations:** ^1^Vaccines Department, Moredun Research Institute, Penicuik, United Kingdom; ^2^Centre for Dementia Prevention, University of Edinburgh, Edinburgh, United Kingdom; ^3^Department of Microbiology and Immunology, University of Western Ontario, London, ON, Canada; ^4^School of Applied Sciences, Napier University, Edinburgh, United Kingdom; ^5^Triveritas Ltd., Brampton, United Kingdom; ^6^University College London Hospital, London, United Kingdom; ^7^Instituto de Ganadería de Montaña (IGM), León, Spain; ^8^Biomathematics and Statistics Scotland, Edinburgh, United Kingdom; ^9^The Roslin Institute and Royal (Dick) School of Veterinary Studies, University of Edinburgh, Midlothian, United Kingdom

**Keywords:** ovine, major histocompatibility complex, indoleamine 2,3 dioxygenase-1, synepitheliochorial placentation, trophoblast

## Abstract

Successful mammalian pregnancies are a result of complex physiological, endocrinological, and immunological processes that combine to create an environment where the mother is tolerant to the semi-allogeneic fetus. Our knowledge of the mechanisms that contribute to maternal tolerance is derived mainly from human and murine studies of haemochorial placentation. However, as this is the most invasive type of placentation it cannot be assumed that identical mechanisms apply to the less invasive epitheliochorial placentation found in other species such as ruminants. Here, we examine three features associated with reproductive immune regulation in a transformed ovine trophoblast cell line and *ex-vivo* ovine reproductive tissues collected at term, namely: major histocompatibility complex (MHC) expression, Indoleamine 2,3 dioxygenase-1 (IDO-1) expression, and Natural Killer (NK) cell infiltration. High levels of MHC class I protein expression were detected at the surface of the trophoblast cell line using a pan-MHC class I specific monoclonal antibody. The majority of MHC class I transcripts isolated from the cell line clustered with classical MHC alleles. Transcriptional analysis of placental tissues identified only classical MHC class I transcripts. We found no evidence of constitutive transcription of IDO-1 in either the trophoblast cell line or placental tissues. *Ex-vivo* tissues collected from the materno-fetal interface were negative for cells expressing NKp46/NCR1. Collectively, these observations suggest that the relatively non-invasive synepitheliochorial placentation found in sheep has a more limited requirement for local immunoregulation compared to the more invasive haemochorial placentation of primates and rodents.

## Introduction

Much of our current knowledge of mammalian reproductive immunology is derived from studies in primates and rodents that share a haemochorial placenta. Haemochorial placentation is a highly invasive process resulting in direct contact between the fetal trophoblast and the maternal decidua and blood (1). In contrast, sheep, and other ruminants have synepitheliochorial placentation, where the uterine epithelium remains largely intact, resulting in a greater separation between the maternal and fetal cell layers. However, these layers are interspersed with syncytial plaques representing fusion of maternal and fetal cells which is unique to ruminants (2). There are a number of reports describing lymphoid subpopulations, cytokines, and expression of major histocompatibility complex (MHC) molecules in the upper reproductive tract of pregnant and non-pregnant sheep (3–8). One of the roles of MHC expression in the human hemochorial placenta is to interact with uterine NK cells and induce expression of angiogenic mediators that improve the blood supply to the developing fetus (1). Relatively little is known about the expression of these molecules and cells in the synepitheliochorial placentation in sheep, a reflection of the paucity of ovine immunological reagents to define these interactions [reviewed by (9)].

We have an ongoing interest in identifying immunoregulatory mechanisms at the maternal-fetal interface in sheep to help us understand the pathogenesis of infectious ovine abortion. *Chlamydia abortus (C. abortus)* is an obligate intracellular Gram-negative bacterium that infects trophoblast and causes abortion in most sheep-rearing countries worldwide (10). *C. abortus* is auxotrophic for tryptophan, hence growth is restricted in cells induced to express the tryptophan-degrading enzyme indoleamine 2,3 dioxygenase-1 (IDO-1). This creates an apparent paradox as placental trophoblast have been reported to constitutively express IDO-1 (11). Until now we have lacked the technical capability to investigate this in more detail. Here we report on the transcriptional expression of MHC class I and IDO-1 in ovine placental tissues collected at full term and the ovine AH-1 trophoblast cell line [derived close to full-term, immortalized, and characterized by Haldorson et al. (12)] and the presence of NKp46/NCR1+ve cells in ovine placental tissue in comparison to what is known for haemochorial placentation.

## Materials and Methods

### Animals and Tissues

Placental tissues (placentome and inter-cotyledonary membrane) were recovered along with the maternal lymph nodes draining the pregnant uterus (lumbo-aortic and medial iliac) and the extra-uterine right pre-femoral lymph node were recovered from seven Dorset-cross ewes at post-mortem at full term of gestation. Placental tissues were stored in RNA*later* (Ambion Life Technologies Europe, Bleiswijk, Netherlands), or snap frozen into super cooled 2-methylbutane (Sigma-Aldrich, Dorset, UK) prior to storage at −70°C. Mesenteric lymph nodes from two 12-month-old Gray-faced sheep experimentally infected with 150,000 *Nematodirus battus* larvae 1 week prior to collection at post-mortem. Venous blood was collected into heparinized vacutainers (Becton–Dickinson, Oxford, UK) and peripheral blood mononuclear cells (PMBC) were isolated by density centrifugation using established protocols (13). All animal procedures were approved by the local Animal Welfare Ethical Review Body and were compliant with the UK Animal (Scientific Procedures) Act 1986.

### Isolation of RNA and Generation of cDNA

Placental lysates were prepared from 30 mg of tissue using a Precellys homogenizer (Bertin Instruments, Basingstoke, UK) operated at 6,000 × g for 30 sec. Each sample was subjected to two rounds of homogenization separated by an incubation of 2 min on ice. Total RNA was isolated using the Qiagen RNeasy® Plus kit (Qiagen Inc., Manchester, UK), which included an on-column DNase digestion to remove any contaminating genomic DNA, in place of using the gDNA eliminator columns, but otherwise following the manufacturer's instructions. The concentration of RNA was determined using a Nanodrop spectrophotometer (Thermo Fisher Scientific, Salt Lake City, USA) and RNA integrity was assessed using a 2100 Bioanalyzer (Agilent Technology, Santa Clara, USA). RNA integrity number (RIN) values were >7.5 for all tissue samples. First stand cDNA was prepared using the ImProm-II Reverse Transcription System (Promega, Madison, USA) using Oligo dT primers in a 40 μL reaction and using 200 ng of RNA.

### Tissue Culture and Preparation of Cells for Flow Cytometry

Ovine AH-1 trophoblast cells were sub-cultured in Iscove's Modified Dulbecco's Medium (IMDM) supplemented with 10% heat-inactivated fetal bovine serum (ΔHFBS, PAA Gold, USA origin, PAA, Hanninger, Austria) and 2 mM L-glutamine (Sigma-Aldrich) (culture medium) in a humidified incubator at 37°C under 5% CO_2_ for 24 h. Cells were harvested by trypsinization to establish single-cell suspensions for flow cytometry or lysed directly within the flasks for RNA isolation by addition of 700 μL of RLT lysis buffer (Qiagen RNeasy kit) containing 0.1% beta-mercaptoethanol (Sigma-Aldrich).

### Detection of MHC Class I Expression on Ovine AH-1 Trophoblast Cells by Flow Cytometry

AH-1 cells were re-suspended to a concentration of 1 × 10^5^ ml in PBS supplemented with 0.05 % (w/v) NaN_3_ (Sigma-Aldrich), 5% ΔHFBS (flow buffer), and stained for viable cells using the Live/Dead® Fixable Violet Dead Cell Stain (Invitrogen, Thermo Fisher Scientific) prior to labeling with the ovine MHC class I specific monoclonal antibody (mAb) IL-A88 at a 1:2 dilution of hybridoma tissue culture supernatant (14). This antibody has previously been shown to bind surface expressed recombinant ovine MHC class I on transfected COS-7 cells (15). Freshly-isolated ovine PBMC were stained as a positive control sample for the flow cytometry. A Border Disease Virus (BDV)-specific mAb VPM 20 was used as an isotype-matched control (IgG2a) at the same 1:2 dilution of hybridoma tissue culture supernatant (16). Cells were incubated for 30 min at 4°C, washed three times in flow buffer prior to addition of 100 μL (1:2,000 dilution) of goat anti-mouse IgG conjugated to Alexa Fluor 488 (Invitrogen). Following a 30 min incubation at 4°C, cells were washed twice in flow buffer and once in PBS, fixed in 200 μL of 1% (w/v) paraformaldehyde (Sigma-Aldrich) in PBS and stored at 4°C in the dark prior to acquisition by flow cytometry.

Between 20,000 and 130,000 events were acquired by a MacsQuant flow cytometer and analyzed using the MacsQuantify Software v2.7 (Miltenyi Biotech, Bergisch Gladbach, Germany) using the gating strategy detailed in Supplementary Figure 1. Briefly, artifacts in acquisition were removed with gating P1, then the main cell population was selected using Side Scatter-Area (SSC-A) vs. Forward Scatter (FSC)-A in P1/P2 gate. Live, viable cells were then selected using VioBlue-A channel vs. FSC-A in the P1/P2/P3 gate and doublet cells excluded in gate P1/P2/P3/P4. The region boundaries were set on the fluorescein isothiocyanate (FITC) channel vs. FSC-A for cells stained with the isotype-matched control mAb. Profiles are displayed as overlayed histogram plots equivalent to above and below the region boundary line of plot E and exactly as shown in plot F of Supplementary Figure 1.

### Defining the Range of MHC Class I Transcripts in the AH-1 Trophoblast Cell Line, Placenta, and Lymph Nodes

MHC class I transcripts were amplified from cDNA prepared from RNA extracted from the AH-1 cell line and lymph node and fetal placental tissues from two of the animals described in section Animals and tissues. Primers 416 and 409 (14) amplify a 500 base pair (bp) fragment which is common to all ovine class I loci and covers the highly polymorphic region between exons 1 and 4. Primers Cfl and DR1 (17) amplify full-length class I transcripts. Together, the majority of the class I transcripts present in the target sample should be amplified. Cycling conditions for the internal fragment included, 94°C for 4 min followed by 30 cycles of 94°C for 30 s, 55°C for 30 s, and 72°C for 30 s. The reaction mix included GoTaq Flexi buffer (Promega), 200 nM of each primer, and 4 μL of a 20 μL reverse transcription reaction as template in 50 μL reactions. Conditions for amplification of the full-length transcripts included an initial denaturation for 5 min, followed by 35 cycles of 94°C for 1 min, 55°C for 30 s, 68°C for 1 min followed by a 5 min extension at 68°C. Amplicons were gel-purified using the SV Gel and PCR clean up Kit (Promega) and cloned into pGEM-T Easy vector (Promega). Twenty colonies per transformation were PCR screened for an insert and 18–20 positive clones were selected for bidirectional Sanger sequencing (Eurofins Genomics, Ebersberg, Germany) with vector-specific T7 and SP6 primers.

### Sequence Analysis

For each clone the forward and reverse sequences were aligned using the SeqMan Pro programme within the DNASTAR Lasergene 14 package and a consensus sequences of each allele was generated from a minimum of three independent clones. Each consensus sequence was screened against a public database of known ovine class I sequences retrieved from the ImmunoPolymorphism Database (IPD)-MHC, [https://www.ebi.ac.uk/ipd/mhc/, Maccari et al. (18)]. This database contains 27 full-length class I transcripts. Four of the sequences are considered non-classical based on a truncated cytoplasmic domain, low levels of transcription in PBMC, and low levels of polymorphism as previously published (15, 17).

### Phylogenetic Analysis

An alignment of the MHC class I nucleic acid sequences generated here along with previously published ovine class I sequences within the IPD-MHC archive was generated using the CLUSTAL Omega software on the EMBL-EBI website (http://www.ebi.ac.uk/Tools/msa/clustalo/). The nucleotide alignment (Supplementary Figure 2) was used to estimate the maximum likelihood phylogenetic tree. Prior to phylogenetic tree estimation, the optimum substitution model was selected using the model selection feature (19) in IQ-TREE (20). The optimum substitution model selected for the class I alignment was TVM+F+I+G4. The topology of the tree was tested with 1,000 bootstrap replicates using the ultrafast booststrap method of Minh et al. (21).

### Development of a Quantitative Real-Time PCR Method to Measure IDO-1 Transcription

The ovine adenocarcinoma cell line ST-6 (22) has previously been shown to up-regulate transcription of the IDO-1 gene following treatment with recombinant ovine (rOv) IFN-γ (11). The biological activity of the rOvIFN-γ preparation [conditioned medium from transfectant Chinese hamster ovary (CHO) cells] was confirmed by viral inhibition assay as previously described (23). Complimentary DNA template from these stimulated cells was used to derive the full length IDO-1 sequence (EU484569.1) and in turn to design the following real time-quantitative (RT-qPCR) Taqman primers and probe: The TaqMan primer ovine IDO-1 RT fwd (5′-CAG AAG GCA CTG CTT GAC ATA TCT-3′), ovine IDO-1 RT rev (5′-GGT TTG GGT CCA CAT ATT CAT GA-3′), and ovine IDO-1 probe (5′-FAM-CCA GCC TGC GCA AAG CCA A-TAM-3′, amplicon size 85 bp). The Prism 3 software (24) was used to design the primers and probe.

The IDO-1 RT-qPCR was optimized as a duplex reaction in conjunction with the 18S rRNA gene using the commercial Eukaryotic 18S primer/ probe RT-qPCR kit (Applied Biosystems, Thermo Fisher Scientific, Cramlington, UK). The IDO-1 and 18S RT-qPCR primer and probe sets were used together in the same well of a 96 well PCR plate (Axygene, Costar, Corning) using a total reaction volume of 25 μL containing: 12.5 μL of 2 × TaqMan Universal PCR mastermix (Applied Biosystems), 2.5 μL of 900 nm IDO-1 Fwd primer, 2.5 μL of 900 nm of IDO-1 Rev primer, 2.5 μL of 2.5 μm IDO-1 probe, 1.25 μL of Eukaryotic 18S primer/probe mix, 2.5 μL of RNase-free water, and 1.25 μL of cDNA (50 ng template) per reaction per well. The positive control sample (cDNA from ST-6 cells stimulated with 250 biological units (bU)/ml rOv IFN-γ for 24 h) was prepared in a Log_2_ dilution series. Control cDNA from unstimulated ST-6 cells and PCR grade water alone served as appropriate non-template controls (NTC). Three technical replicates of each sample/standard, as a measure of intra-sample consistency, were amplified using the thermal cycling conditions of 50°C for 2 min, 95°C for 10 min, and 45 cycles of 95°C for 15 s, and 60°C for 1 min using the ABI 7000 Thermocycler (Thermo Fisher Scientific).

Analysis of the optimisation standard curves of the slope of the Log_10_ Concentration vs. Cycle Threshold (Ct) values were approximately equal for both targets (ovine IDO-1 −3.67 and 18S −3.60) consistent with the recommended Minimum Information for Publication of Quantitative Real-Time PCR Experiments (MIQE) Guidelines set out by Bustin et al. (25). A titration of a positive control sample using a Log_4_ dilution series revealed a constant delta (Δ) Ct (IDO-1 Ct−18S Ct) for the entire range when the RT-qPCR was run in a duplex reaction.

### Quantifying IDO-1 Expression in the AH-1 Trophoblast Cell Line, Placental Tissues, and Extra-Uterine and Uterine Lymph Nodes

AH-1 cells were sub-cultured in 24 well-flat bottom plates at a cell density of 1 × 10^5^ cells/ well in a total volume of 1 ml. After 24 h, the culture media was replaced with IMDM supplemented with 2% ΔHFBS and 2 mM L-glutamine (maintenance medium) containing different doses of rOv IFN-γ (1, 10, and 100 bU/ml). Wells containing an equivalent dilution of conditioned medium derived from the untransfected CHO cell line (UTF) acted as unstimulated controls. Each treatment was set up in duplicate wells. After a 48 h incubation period, cells from duplicate wells were harvested into lysis buffer, pooled to provide one sample per treatment, and RNA prepared as described previously. cDNA was prepared using the Applied Biosystems Reverse Transcription Kit using random hexamers to prime the reverse transcription reaction. Each RT reaction included: 5.0 μL of 10 × RT buffer, 8.8 μL MgCl_2_, 8 μL dNTPs, 2.0 μL random hexamers, 1.0 μL reverse transcriptase, 0.8 μL RNase inhibitor, and 1 μg of RNA and water to 50 μL. The reaction conditions were: 25°C for 10 min, 48°C for 1 h, and 95°C for 5 min.

cDNA was also prepared from the ovine placental and lymph node RNA samples using the Applied Biosystems kit as detailed above. The duplex RT-qPCR reaction included 50 ng of cDNA in triplicate test wells for each sample. The AH-1 trophoblast data was analyzed (setting of thresholds and baselines) using the ABI 7000 software Sequence Detection Software (S.D.S.) version 1.2.3 and presented using the comparative Ct (2^−ΔΔCt^) method (26). The relative difference in IDO-1 expression between AH-1 cells stimulated with different doses of IFN-γ was determined using AH-1 cells exposed to UTF conditioned medium as the calibrator sample. Experiments were carried out on four separate occasions with the exception of the IFN-γ treatment at 1 bU/ ml where this was undertaken twice. The ovine tissue sample data was analyzed using the S.D.S. software and presented using the comparative Ct (2^−ΔCt^) method (27).

### Detection of NKp46/NCR1+ve Cells in Ovine Placental Tissues by Immunohistochemistry

The snap frozen mesenteric lymph node tissues from *N. battus-*infected ewes were used as a source of tissue known to be positive for cells expressing NKp46/NCR1 (28). The mesenteric lymph node from the parasite-infected animals and the uterine and extra-uterine tissue samples collected from seven female Dorset-cross sheep at full-term gestation were sectioned by Cryostat (8 μm) and fixed in cold methanol (−20°C), then frozen at −80°C prior to mounting using an established protocol (29). The En Vision Plus HRP system (Dako, Agilent Technology) was used to detect the binding of the NKp46/NCR1 molecule within the tissue sections according to the manufacturer's instructions. Briefly, fixed slides were loaded into a Sequenza staining chamber system (Thermo Fisher Scientific) and washed twice in PBS. The slides were blocked for endogenous peroxidase using peroxidase block for 20 min at room temperature (RT). Slides were then washed twice with PBS before an additional 30 min incubation with 25% normal goat serum (NGS, Vector laboratories, Peterborough, Canada) to block non-specific antibody binding. Culture supernatant of the primary mAb GR13.1, [specific for ovine NKp46/NCR1 as characterized by Connelley et al. (8)] was prepared in-house from the hybridoma and applied to the slides at 1:25 dilution in PBS and incubated overnight at RT. Hybridoma culture supernatant of a negative control isotype-matched (IgG1) BDV-specific mAb VPM 21 (16) was used at 1:25 dilution. Slides were then washed twice with PBS prior to the application of the goat anti-mouse IgG1-horse radish peroxidase (HRP) secondary antibody applied at 1:1,000 in PBS for 30 min at RT. Slides were washed twice prior to the labeling of cells by application of the substrate chromogen (DAB chromogen diluted 1:50 in substrate buffer) for 8 min.

The slides were then washed with distilled water and counter-stained with haemotoxylin (Cellpath, Newton, Wales), blued-up with Scott's tap water substitute, dehydrated, cleared, and mounted using consul-mount (Thermo Fisher Scientific, Cheshire, UK). Slides with the negative controls were included to confirm specificity, namely isotype-matched control mAb, no primary antibody, and DAB only controls.

### Statistical Analyses

Overall differences between treatment groups (rOv IFN-γ at doses 1, 10, and 100 bU/ ml) in mean AH-I IDO-1 RT-qPCR fold change relative to the control group (UTF treatment group) from four independent experiments were statistically tested in log_10_-scale using a one-way Analysis of Variance (ANOVA) model. *Post-hoc* pair-wise comparisons between treatment groups were conducted from the ANOVA model estimates, with the corresponding *p*-values adjusted to control for false discovery rate (FDR). The direct statistical comparisons of AH-I IDO-1 RT-qPCR values between individual treatments and control group were determined by *t*-tests of the mean log_10_-fold change against zero. Statistical test significance was assessed at the usual 5% significance level.

## Results

### The Ovine AH-1 Trophoblast Cell Line Expresses High Levels of MHC Class I

**Figure 1:**
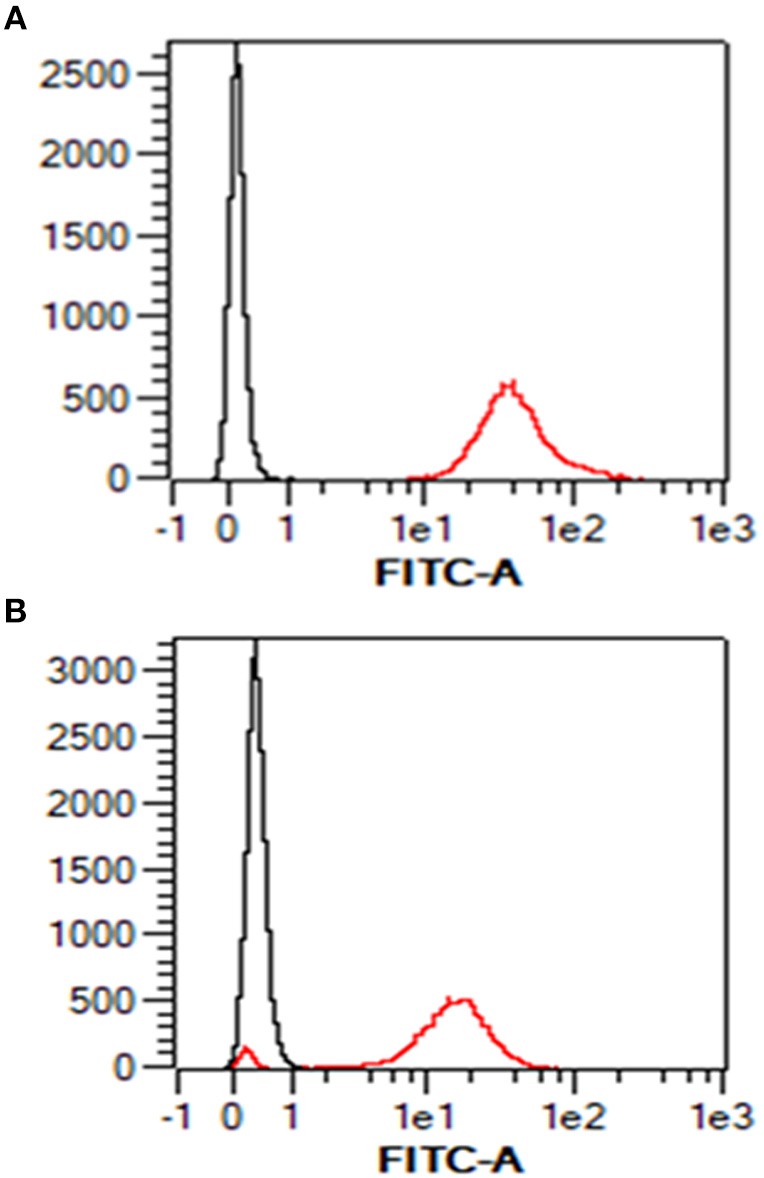
Surface staining of MHC class I in ovine PBMC and ovine AH-1 trophoblast cell line detected by flow cytometry. Ovine peripheral blood mononuclear cells (PBMC) were prepared as described in section Animals and Tissues and the ovine AH-1 trophoblast cultured as described in section Tissue Culture and Preparation of Cells for Flow Cytometry. The cells were prepared for flow cytometric analysis as described in section Tissue culture and preparation of cells for flow cytometry and labeled for flow cytometric analyses in section Detection of MHC Class I Expression on Ovine AH-1 Trophoblast Cells by Flow Cytometry. Cells were acquired using the MacsQuant flow cytometer and analyzed using the MacsQuantify Software v2.7 using the following gating strategy displayed and described in full (Supplementary Figure 1). Ovine PBMC is represented in **(A)** (upper panel) and the ovine AH-1 trophoblast cell line in **(B)** (lower panel) in overlaying histogram plots derived from the display and described in full in Supplementary Figure 1 where the black line represents cells stained with the isotype-matched control mAb against Border Disease Virus and red line represents the cells stained for MHC class I.

Given the absence of any prior knowledge of MHC expression on the surface of the AH-1 cell line, we validated our staining protocol on freshly-isolated ovine PBMC using the pan-ovine MHC class I-specific mAb IL-A88. As expected, all PBMC stained positive with IL-A88 (Figure 1A, red line) in comparison to the isotype-matched negative control mAb VPM 21 (Figure 1A, black line). Surface MHC class I expression was detected on over 90% of the AH-1 cells (Figure 1B, red line) compared with the isotype-matched control (Figure 1B, black line).

**Figure 2:**
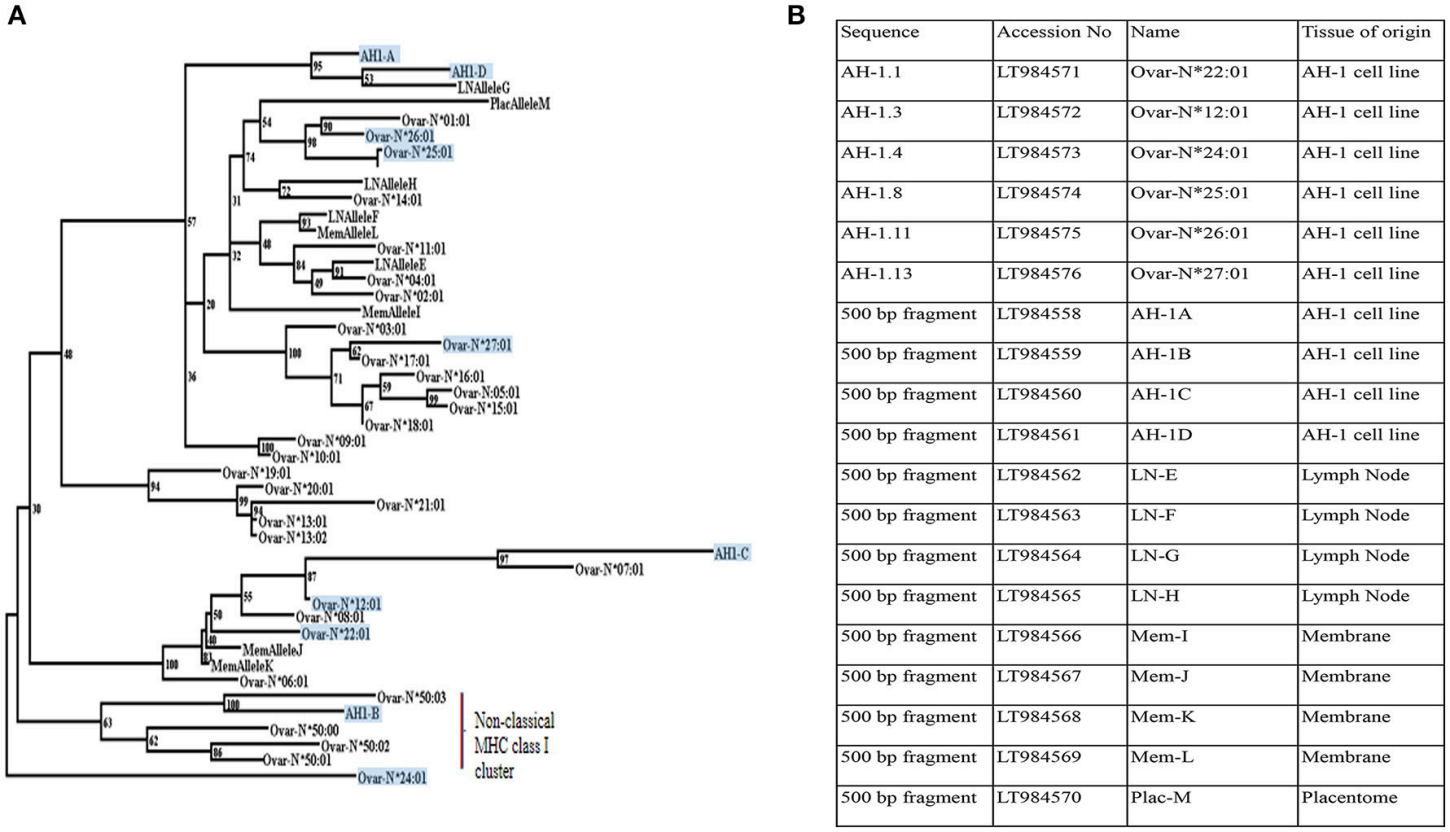
Phylogenetic reconstruction of the relationships between ovine MHC class I transcripts. **(A)** Phylogenetic relationships between MHC class I transcripts identified in this study with published sequences in Ovar IPD-MHC database. Maximum likelihood phylogenetic analysis of the relationships between MHC class I nucleotide sequences identified in this study and all those archived in the IPD-MHC Database. The tree is based on the alignment shown in Supplementary Figure 2. Details of each of the novel transcripts identified in this study are listed in Figure 2B. All transcripts derived from the AH-1 trophoblast cell line are boxed and shaded. Transcripts derived from the lymph node are assigned LN Allele E to H. MemAllele I to L are derived from inter-cotyledonary membrane and PlacAllele M is derived from placentome. **(B)** Novel full-length and internal MHC class I sequences derived from the ovine AH-1 trophoblast and placental tissues and selected maternal lymph nodes at full term of gestation. A list of unique ovine MHC Class I transcripts identified in this study listed with Accession numbers, sequence names, and the tissue of origin.

The pan-specific nature of IL-A88 is useful for detecting all expressed MHC class I molecules. As no mAbs are available to discriminate between classical and non-classical MHC class I molecules in sheep, we therefore employed transcriptional analyses to determine the nature of the MHC class I molecules expressed by the AH-1 cells. Ten distinct MHC class I transcripts were amplified from the AH-1 cell line using primers described in section Defining the range of MHC class I transcripts in the AH-1 trophoblast cell line, placenta, and lymph nodes. The nucleotide sequence of each transcript is shown in Supplementary Figure 2. Of these, only one full-length transcript, Ovar-N^*^12:01 had previously been described. Of the remaining nine sequences, five novel full-length sequences were submitted to the European Nucleotide Archive (ENA) and the IPD-MHC Database to be assigned official names. The remaining four novel internal sequences do not meet the size requirements to receive an official name but were assigned a unique name which reflects the order in which they were identified (Figure 2B).

A BLAST search against the previously published ovine classical and non-classical MHC class I transcripts held within the IPD-MHC Database suggested that only a single transcript derived from the AH-1 trophoblast cell line (AH-1B) was non-classical in nature. This was an unexpected result which could have been a unique reflection of this cell line. We therefore extended our MHC class I analyses to placentome and inter-cotyledonary membrane as well as maternal lymph nodes.

### Analysis of MHC Class I Transcripts in Placentome, Inter-cotyledonary Membrane, and Maternal Lymph Nodes

Primers targeting the internal fragment described in section Defining the range of MHC class I transcripts in the AH-1 trophoblast cell line, placenta, and lymph nodes amplified nine distinct MHC class I transcripts from the placentome, inter-cotyledonary membrane, and maternal lymph node tissues. The nucleotide sequence of each is shown in Supplementary Figure 2. All of these sequences were novel and each was submitted to the ENA. Database accession numbers are listed in Figure 2B along with the sequences from the AH-1 cells. The predicted amino acid sequences of all of the transcripts described in Figure 2B are shown in Supplementary Figure 3. A Nucleotide BLAST search against the previously published ovine classical and non-classical MHC class I transcripts held within the IPD-MHC Database (shown in Supplementary Figure 2) suggests that all transcripts derived from the placentome, inter-cotyledonary membrane, and maternal lymph node tissues were classical in nature.

### Phylogenetic Analysis

To further analyse the sequences derived from this study, a maximum likelihood phylogenetic tree was constructed from an alignment of the ovine MHC class I transcripts held in the IPD-MHC database (https://www.ebi.ac.uk/ipd/mhc/) alongside those identified from this study. The tree shown in Figure 2A indicates that the majority of the transcripts derived from placental tissues, the AH-1 cell line, and the maternal LN all clustered with other classical MHC class I sequences. Only a single sequence derived from the AH-1 trophoblast cell line (AH-1B) clustered with the other non-classical class I sequences.

### IDO-1 Expression in the Ovine AH-1 Trophoblast Cell Line, Placental Cotyledon, and Intercotyledonary Membranes

**Figure 3:**
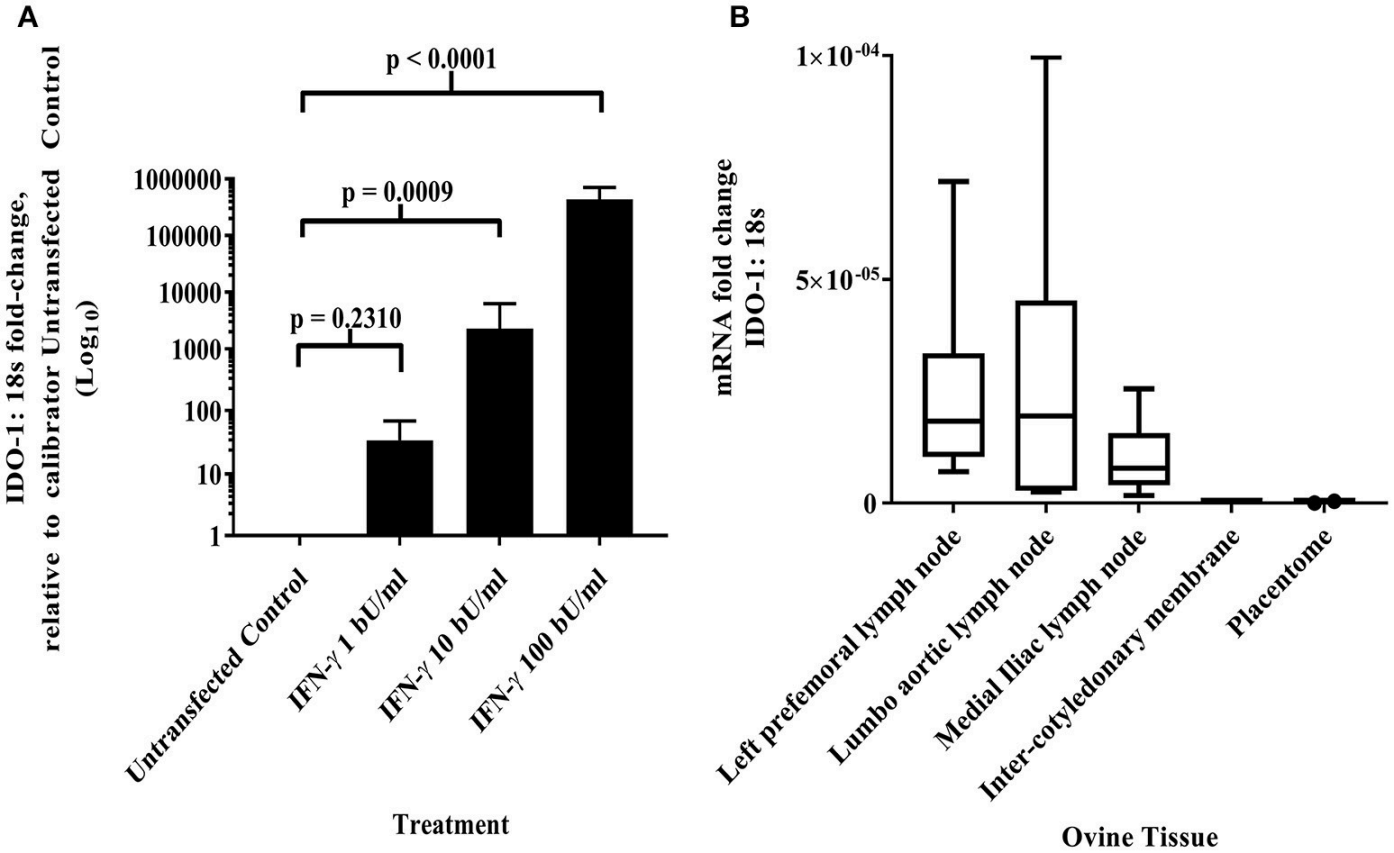
IDO-1 mRNA transcription **(A)** IFN-γ up-regulates IDO-1 transcription in dose-dependent manner in ovine AH-1 trophoblast cell line. Ovine AH-1 trophoblast cells were cultured in a 24 well-plate as previously described in section Quantifying IDO-1 Expression in the AH-1 Trophoblast Cell Line, Placental Tissues and Extra-Uterine, and Uterine Lymph Nodes with maintenance medium supplemented with rOv IFN-γ (1, 10, and 100 biological units (bU)/ ml) or control untransfected conditioned medium alone for 24 h. Cell lysates were collected for down-stream processing to cDNA prior to running on the duplex qRT-PCR for IDO-1 and 18S (described in detail in section Quantifying IDO-1 Expression in the AH-1 Trophoblast Cell Line, Placental Tissues, and Extra-Uterine and Uterine Lymph Nodes). Data has been transformed using the comparative Ct (2^−ΔΔCt^) method (26) using AH-1 + UTF sample as the calibrator in line with the MIQE guidelines (25). Each bar represents the arithmetic mean of four experiments (except for AH-1 + 1 bU/ml rOv IFN-γ treatment, which was run twice) and the error bars represent the standard error. Statistical analyses has been described in detail in section Statistical analyses using a one-way ANOVA. **(B)** IDO-1 transcription is differentially expressed in lymph nodes and absent placental tissues. Ovine placental and lymph node tissues were collected from seven sheep as described in sections Animals and tissues and subsequent processing to cDNA as described in detail in section Isolation of RNA and generation of cDNA. The duplex IDO-1 and 18S qRT-PCR was performed as described in sections Development of a Quantitative Real-Time PCR Method to Measure IDO-1 Transcription–Quantifying IDO-1 Expression in the AH-1 Trophoblast Cell Line, Placental Tissues, and Extra-Uterine and Uterine Lymph Nodes. The data have been transformed using the comparative Ct (2^−ΔCt^) method. The mRNA fold-change data from the seven ewes has been presented in the box and whisker plots for each tissue (right-prefemoral, lumbo-aortic, and medial iliac lymph nodes; inter-cotyledonary membrane and placentome). The center line displays the arithmetic mean and the box represents the 10th and 90th percentiles. The whiskers show the spread of the data.

Maternal CD8+ve T cell responses to paternal MHC class I can be regulated by constitutive fetal trophoblast expression of IDO-1 in murine allogeneic matings (30). Given the predominance of classical MHC class I expression by the AH-1 cell line and placental tissues, we were interested in knowing if IDO-1 was expressed at the materno-fetal interface in sheep. Using the ovine IDO-1 RT-qPCR assay described in section Development of a quantitative real-time PCR method to measure IDO-1 transcription, no constitutive transcription of the IDO-1 gene was detected in the AH-1 trophoblast cell line. However, IDO-1 transcription was induced following treatment with rOv IFN-γ in a dose-dependent manner (Figure 3A, overall ANOVA test *p* < 0.0001), with 10 and 100 bU/ ml being statistically resulting in expression significantly higher than UTF-treated cells (UTF control cells, *p* = 0.009 and *p* < 0.0001, respectively). Analysis of placentome and inter-cotyledonary membranes collected from seven ewes at term revealed no constitutive transcription of the IDO-1 gene (Figure 3B). In contrast, IDO-1 transcription was detected in uterine and extra-uterine lymph nodes, with notable variation between animals, and also between lymph nodes from the same and different animals (Figure 3B).

### NKp46/NCR1 Expression in Cells at the Ovine Materno-Fetal Interface

**Figure 4:**
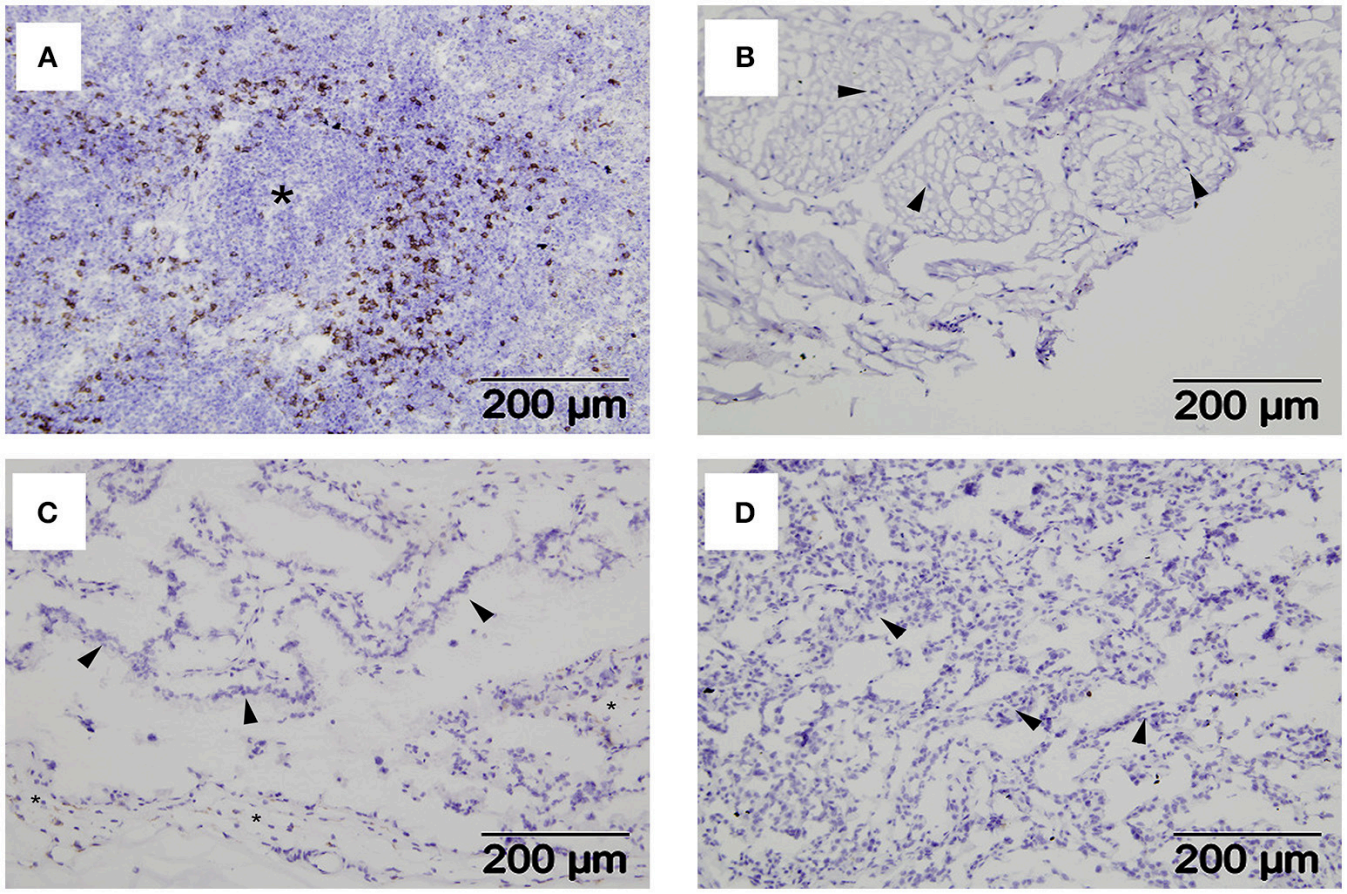
Distribution of NKp46+ve cells within extra-uterine lymph nodes and placental tissues. Representative frozen sections of lymph node and placental tissues collected at post-mortem from seven pregnant ewes plus lymph nodes from two ewes infected with *Nematodirus battus* as positive control inflamed lymph nodes (described in detail in section Animals and Tissues). Serial sections were labeled with a mAb specific for ovine NKp46/NCR1 or an isotype-matched control mAb against Border Disease Virus using the immunohistochemical technique described in section Detection of NKp46/NCR1+ve Cells in Ovine Placental Tissues by Immunohistochemistry. NKp46/NCR1+ve cells visualized by brown labeling can be seen in the paracortex around a lymphoid follicle (asterisk) in the *N. battus* infected mesenteric lymph node **(A)**. The isotype-matched control mAb staining for this tissue is shown in Supplementary Figure 4. Tissues from pregnant ewes are shown in images **(B–D)** as follows: **(B)** deep functional zone of the intercaruncular endometrium showing loose connective tissue and fibrocytes (arrowheads); **(C)** chorionic inter-cotyledonary membrane formed by mesenchymal tissue (asterisk) and trophoblast cells (arrowheads); **(D)** interdigitated area of placentome where cells from the branching maternal and fetal villi are present but cannot easily be distinguished, the thicker accumulations of cells (arrowheads) correspond to maternal septa. A summary of the NKp46/NCR1 expression in tissues from the pregnant ewes can be found in Supplementary Table 1.

Interactions between maternal uterine NK cells and fetal trophoblasts expressing classical and non-classical MHC class I molecules are important for immune regulation and angiogenesis during human placentation (31). Given our MHC expression findings, we were also interested in determining if NK cells were also a feature of the materno-fetal interface in sheep. Using a monoclonal against ovine NKp46/NCR1 (8), we initially validated our staining protocol using mesenteric lymph node tissue from a sheep infected with the nematode parasite *N. battus* and found large numbers of NKp46/NCR1+ve cells in the paracortex surrounding the lymphoid follicles (Figure 4A) and absent in the equivalent serial section using the isotype-matched mAb (Supplementary Figure 4). However, no NKp46/NCR1+ve cells were found in the uterine wall (Figure 4B), placental membrane (Figure 4C), or placentome at term (Figure 4D) suggesting that these cells are not an integral feature of normal ovine pregnancy (data summarized in Supplementary Table 1).

## Discussion

Mammalian placentation can be categorized into four major structural types, namely epitheliochorial, syneptheliochorial, endotheliochorial, and haemochorial. These structures have arisen through a process of convergent evolution and vary in the degree of fetal trophoblast invasion into the maternal uterine tissues (2). One of the earliest proposed hypotheses to help explain maternal acceptance of the semi-allogeneic fetus is that the placenta acts as a barrier between mother and fetus (32). While this is partially true, there are also many other immunoregulatory mechanisms involved reviewed by Billington (33). Among these, inflammation appears to be an evolutionarily-conserved feature of the early and late stages of normal mammalian pregnancy (34, 35). We have observed elevated expression of pro-inflammatory mediators such as TNF-α in the placentas of sheep that experience early abortion as a result of *C. abortus* infection and have reproduced these findings in infected AH-1 trophoblast cells (36). We have previously reported that peripheral antigen-specific cellular immune responses in pregnant sheep do not display the Th1-type to Th2-type paradigm shift that is observed in humans and mice (37). At that time, we hypothesized that this could reflect a lower threshold requirement for gestational immune modulation in sheep with less-invasive synepitheliochorial placentation compared to species with highly-invasive haemochorial placentation. However, we had no detailed knowledge of the immunological features at the materno-fetal interface to associate this with local immune regulation in the placenta.

Major advances in reproductive immunology have been made using trophoblast cell lines, perhaps the most notable being the role that the human BeWo choriocarcinoma cell line played in the discovery of HLA-G (38). The AH-1 ovine trophoblast cell line is therefore a valuable resource for sheep reproductive immunology. When this cell line was first developed by SV40 transformation of sheep fetal cotyledon cells it was shown to be positive for ovine IFN-τ and negative for ovine placental lactogen (12). We focused our analysis on this cell line as we speculated that it may allow identification of the ovine equivalent of the non-classical MHC class I molecule HLA-G. We established that this cell line expresses high levels of surface MHC class I (Figure 1B) using a pan-ovine MHC class I-specific mAb, but were unable to further discriminate between classical and non-classical MHC class I at the protein level. Transcriptional analysis revealed 10 MHC class I transcripts, one of which appears to be non-classical in nature, the other nine being classical (Figure 2A). Whether the non-classical transcript represents the ovine equivalent of HLA-G remains to be determined, however the failure to identify it in the placental tissues analyzed here, suggests otherwise.

When we extended our transcriptional analysis to *ex-vivo* tissues derived from placental membrane, placentome, and lymph node collected at term, we only found classical MHC class I sequences (Supplementary Figure 2). The results from the AH-1 cell line suggest that there is the potential for non-classical MHC class I expression in the ovine placenta, but the results from the cell line and the tissues suggest that classical MHC class I expression dominates. This may not be too surprising as both classical and non-classical MHC class I expression are features of human and mouse placentation and components of the normal reproductive processes such as uterine vascularisation. Human placental trophoblasts do not express the highly-polymorphic products of the classical MHC class I HLA-A and—B loci but do express classical HLA-C and non-classical HLA-E and—G, while mouse trophoblasts express products of the classical H2-K locus but not the H2-D locus and lack expression of the products of the non-classical M, Q, and T genes (39). In cattle, which share synepitheliochorial placentation with sheep, placental trophoblast cells express both classical, and non-classical MHC class I, with proportionately more non-classical expression compared to PBMC (40). Collectively, these observations suggest that the roles of classical vs. non-classical MHC class I in normal reproduction are complex, species specific, and cannot be attributed solely to the placental type and degrees of invasiveness.

One of the important functions of trophoblast MHC class I expression in humans and mice is to engage receptors on maternal NK cells, supporting fetal growth by inducing local angiogenesis, and suppressing immune attack (31, 39). NK cells have previously been reported sparsely distributed in the non-gravid sheep uterus using a mAb that detects ovine NKp46/NCR1 (8). Using the same mAb we did not detect NK cells in sheep uterus collected at term, nor did we observe NK cells in placental tissues (placentome and membrane) collected at term (Figures 4B–D). This is not due to a functional failure of the mAb, as we found extensive NK cell staining in mesenteric lymph node from a parasite-infected sheep, localized to the paracortical area surrounding the follicles as expected for the positive control (Figure 4A) (28). The failure to detect NK cells in ovine reproductive tissue could be due to the timing of sample collection as uterine NK (uNK) cell numbers reduce as pregnancy progresses, particularly in the third trimester in humans (41) although the total absence in all tissues from the materno-fetal interface suggests that they are unlikely to be playing a role in ovine pregnancy.

As discussed earlier, the transcription of classical MHC class I genes in tissues at the materno-fetal interface in sheep and the over-dominance of classical MHC class I gene transcription in the ovine trophoblast cell line is not out of line with trophoblast expression of classical polymorphic MHC class I molecules in humans and mice. However, given the potential for activation of maternal T cells by paternal MHC class I, there are a number of immunoregulatory processes operational in haemochorial placentation (42). Among these, constitutive trophoblast IDO-1 expression has been shown to specifically control maternal CD8+ve T cell-mediated rejection of the semi-allogeneic fetus in mice (30) and has also been shown to be constitutively expressed in human trophoblast (43). We found no constitutive IDO-1 expression in the *ex-vivo* ovine placental tissues collected at term or in the AH-1 cell line (Figure 3). However, we did detect IDO-1 transcripts in lymph node tissues serving as a positive control for the assay (Figure 3B). Again, the timing of placental sample collection could have influenced the results. IDO-1 is expressed at higher levels in early gestation but is still present at parturition in humans (44), hence we would have expected to see some signal if this was true for sheep. Interestingly, we could induce IDO-1 expression in AH-1 cells with rOv IFN-γ (Figure 3A) whereas the same is not true for BeWo cells and recombinant human IFN-γ (45). This has implications for the interpretation of data derived from their applications in host-pathogen studies *in vitro*, particularly pathogens that are controlled by IDO-mediated tryptophan degradation (11).

In summary, we have found notable differences in MHC class I transcription, IDO-1 expression, and NK cell distribution in sheep reproductive tissues compared to humans and mice. We have made these observations in *ex-vivo* reproductive tissues collected at term from pregnant sheep and in a transformed ovine trophoblast cell line. Our data are therefore derived from a single time-point at the end of gestation. Given the changes that occur in expression of immunological mediators throughout pregnancy in other species studied to date, it would be interesting to apply the technologies we have developed in this paper to samples collected throughout ovine pregnancy. While it may be an over-simplification to attribute these differences to the different placental structures between the species, they add to our knowledge of comparative reproductive immunology, and have implications as to how these species control reproductive pathogens.

## Author Contributions

SW, GE, JB, CM, and KB designed the study. SW, GE, and JB collected the tissue samples. SW, DW, NW, CM, and GE undertook the IDO cloning. SW and NW undertook the AH-1 trophoblast IDO-1 expression. SW and JP undertook the IDO-1 tissue analyses. SW, MG, and KB undertook the MHC class I expression. LD, SW, and GE undertook the NK cell analyses. JP-A undertook the statistical analyses of the IDO-1 expression data. SW, CM, KB, and GE drafted the manuscript. All co-authors read and revised the submitted manuscript.

### Conflict of Interest Statement

The authors declare that the research was conducted in the absence of any commercial or financial relationships that could be construed as a potential conflict of interest.
