# Supplementary material for: Immunological Homeostasis at the Ovine Placenta May Reflect the Degree of Maternal Fetal Interaction
Source: Front Immunol. 2019 Jan 9;9:3025. doi: 10.3389/fimmu.2018.03025 (PMC6334339; doi:10.3389/fimmu.2018.03025)
Supplement: Supplementary file 1 [file Data_Sheet_1.docx]

Supplementary Material

**Immunological homeostasis at the ovine placenta may reflect the degree of maternal foetal interaction**

Sean R Wattegedera^1^*, Laura Doull^2^, Mariya Goncheva^3^, Nicholas M. Wheelhouse^4^, Donna M. Watson^5^, Julian Pearce^6^, Julio Benavides^7^, Colin J. McInnes^1^, Keith Ballingall^1^ and Gary Entrican^1,8^

*** Correspondence:** Corresponding Author: [sean.wattegedera@moredun.ac.uk](mailto:sean.wattegedera@moredun.ac.uk)

# Supplementary Figures and Tables

## Supplementary Figure 1 Gating strategy for assessment of MHC Class I specific labelling of ovine PBMC and ovine AH-1 trophoblast

Ovine PBMC and AH-1 cells were prepared for flow cytometric analyses as described in detail in sections Tissue culture and preparation of cells for flow cytometry and Detection of MHC Class I Expression on ovine AH-1 trophoblast cells by flow cytometry. Between 20, 000 and 130, 000 events were acquired by a MacsQuant flow cytometer for each sample and analysed using the MacsQuantify Software v2.7. (Miltenyi Biotech, Bergisch Gladbach, Germany) using the following gating strategy. Artefacts in cell acquisition were removed with gate P1 (Forward Scatter-area (FSC-A) vs High Dynamic Range over time (HDR-T), then the main cell population was selected using Side Scatter-Area (SSC-A) vs FSC-A in P1/ P2 gate. Live, viable cells were then selected using VioBlue-A channel vs FSC-A in the P1/ P2/P3 gate. Doublet cells excluded in gate P1/ P2/ P3/ P4. The region boundaries were set on the fluorescein isothiocyanate-Area (FITC-A) channel vs Side Scatter Area (SSC-A) for cells stained with the isotype-matched control mAb against Border Disease Virus. Overlaying histogram plots of Log_10_ FITC-A using P1/ P2/ P3/ P4, plot F gating strategy equivalent to plot E (above and below the region boundary line).


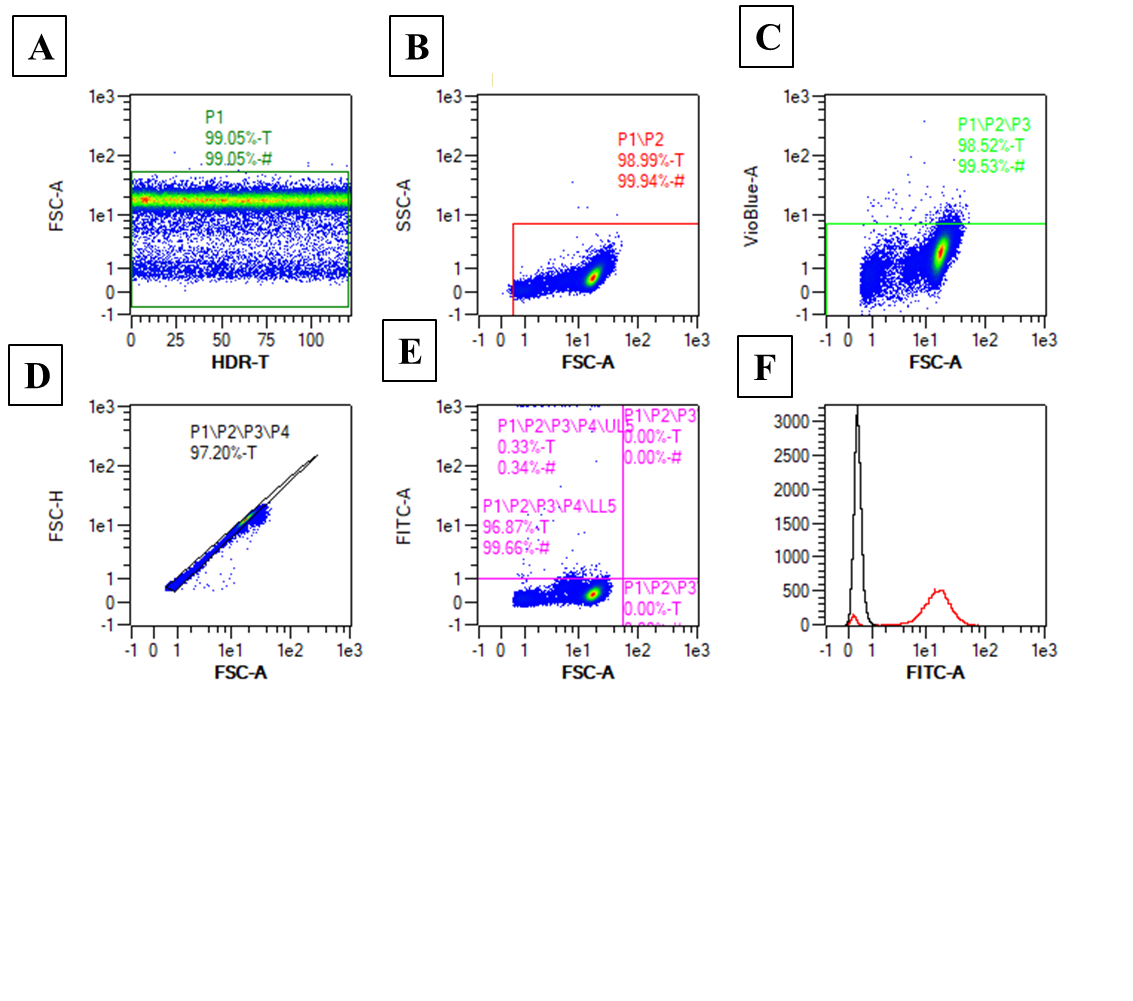


**Supplementary Figure 2 Nucleotide MHC class I alignment of AH-1 trophoblast, ovine lymph node and placental tissue sequences with published IPD-MHC archive sequences.**

An alignment of the class I nucleic acid sequences generated in this study with previously published ovine class I sequences within the IPD-MHC archive was generated using the CLUSTAL Omega software on the EMBL-EBI website (<http://www.ebi.ac.uk/Tools/msa/clustalo/>). Ova-N*01:01 transcript from the IPD database has been used as the consensus sequence. Sequences derived from the AH-1 trophoblast, lymph node, inter-cotyledonary membrane and placentome are defined with the prefix as follows: AH1, LN, Mem and Plac respectively.


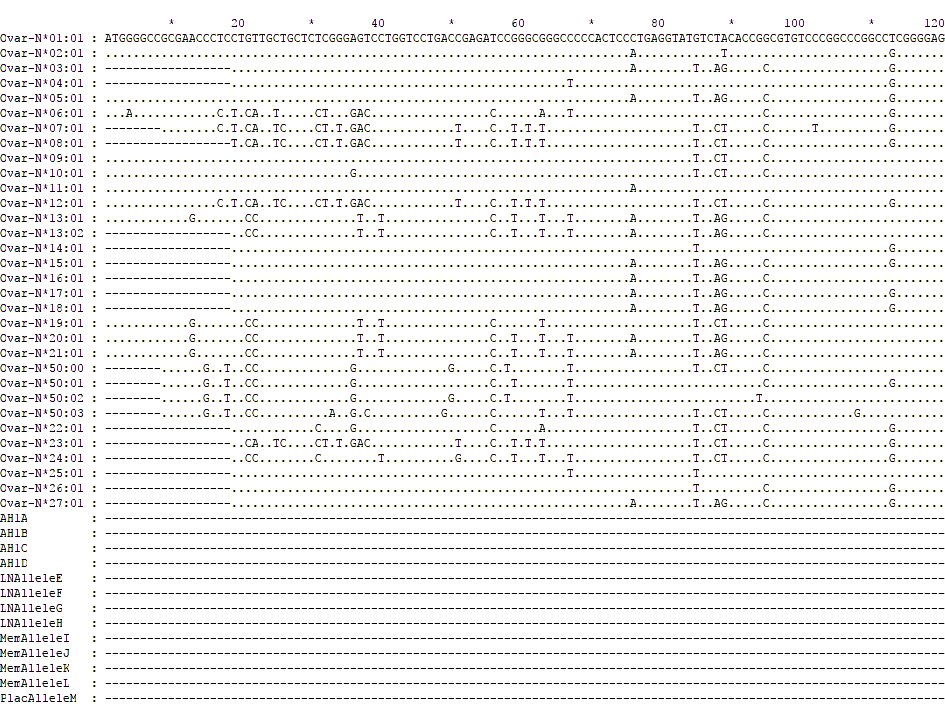


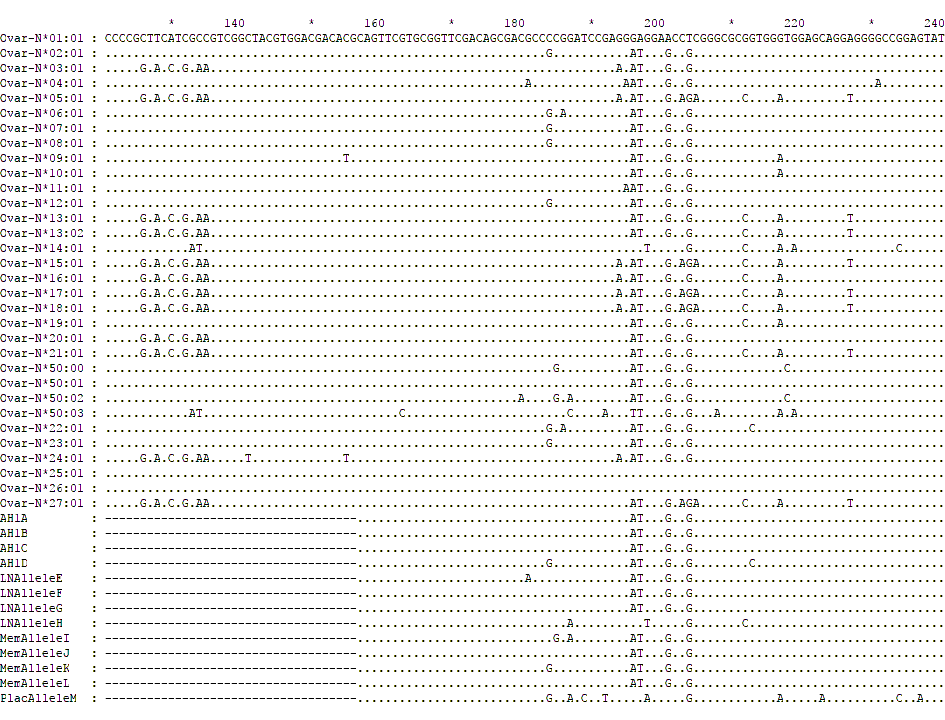


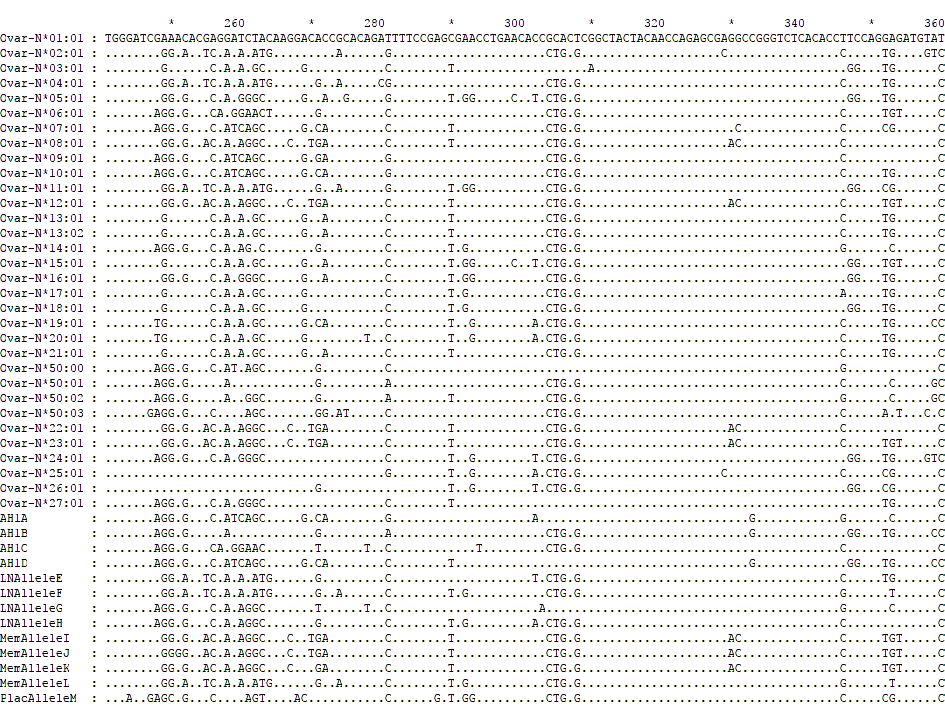


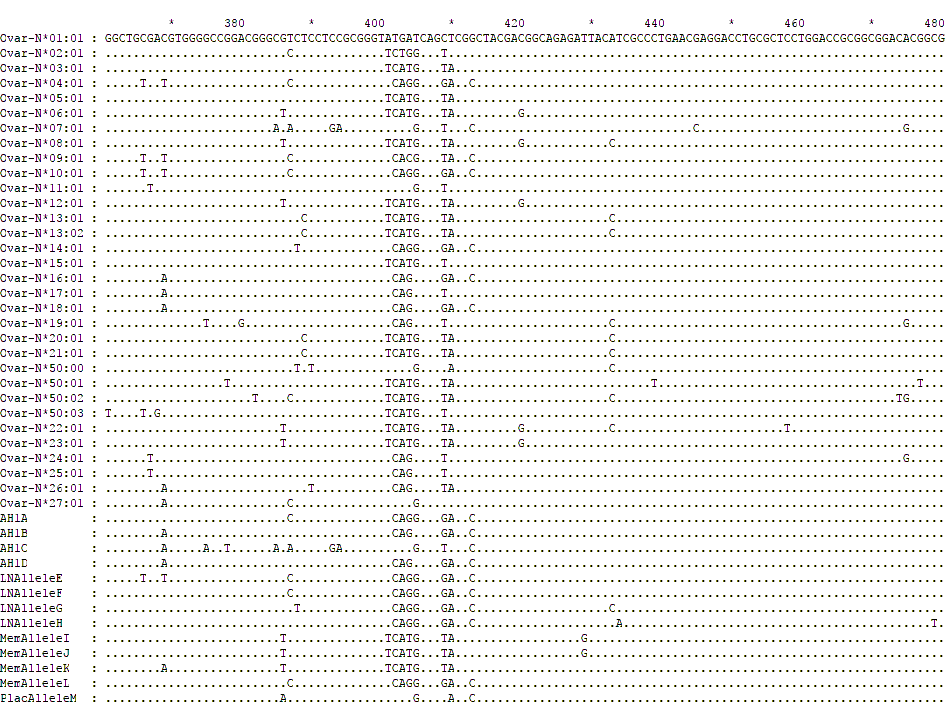


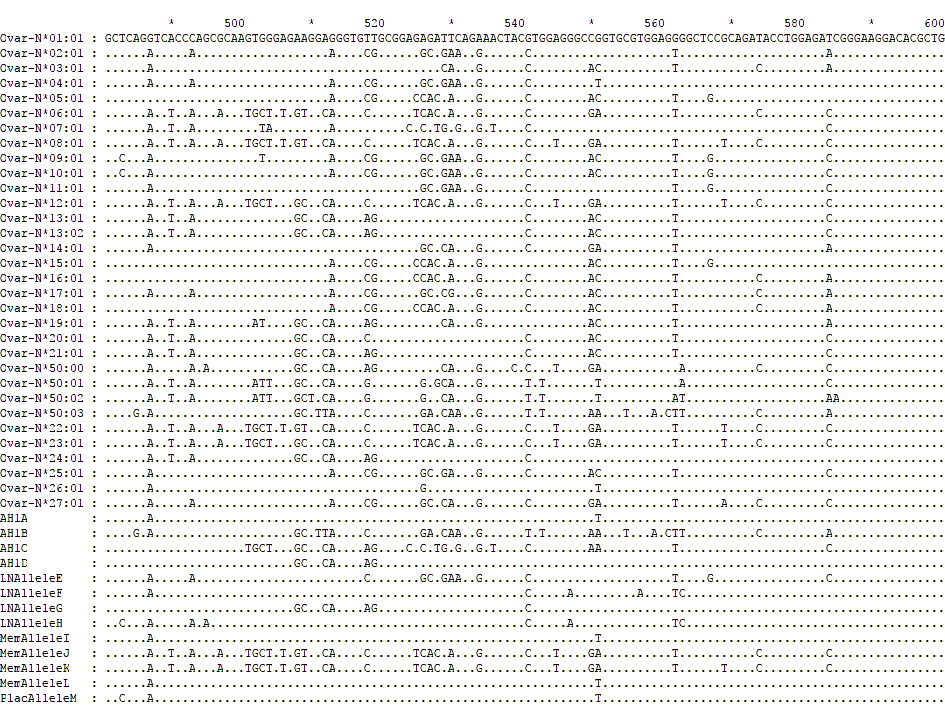


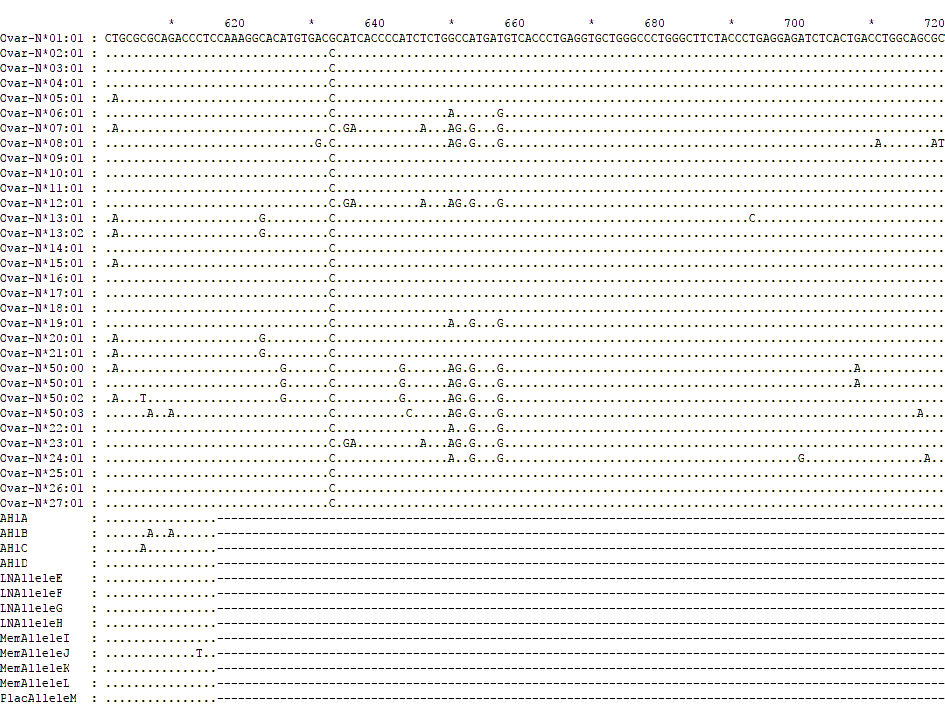


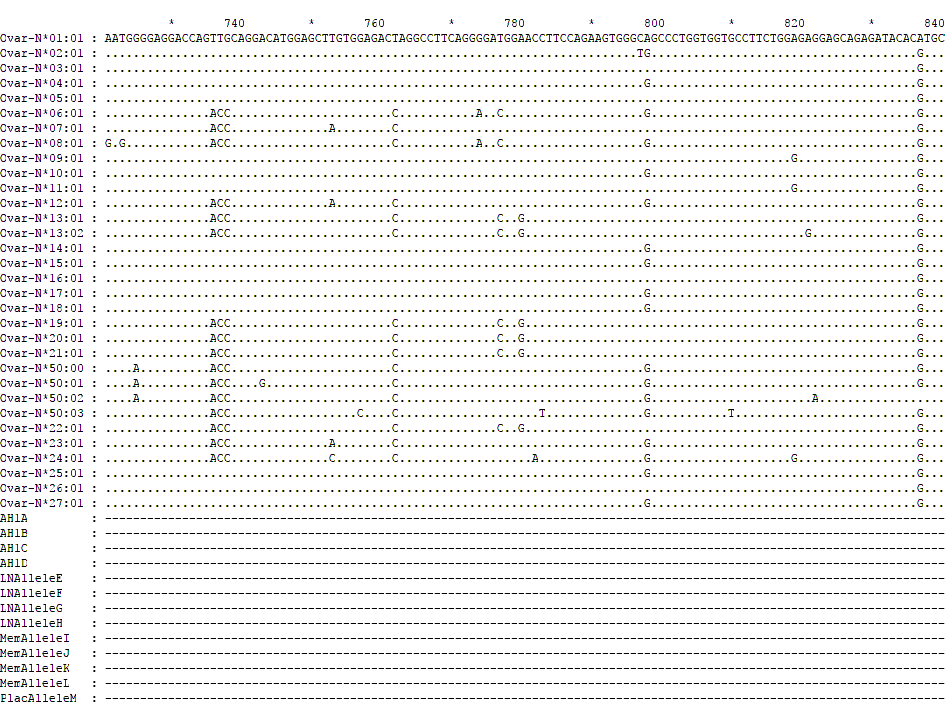


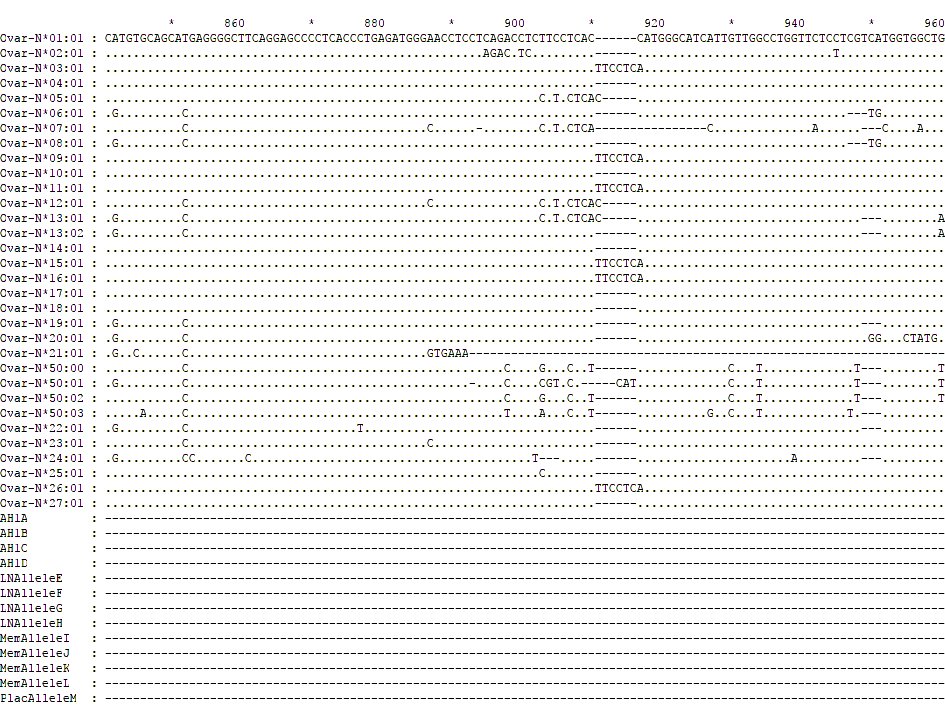


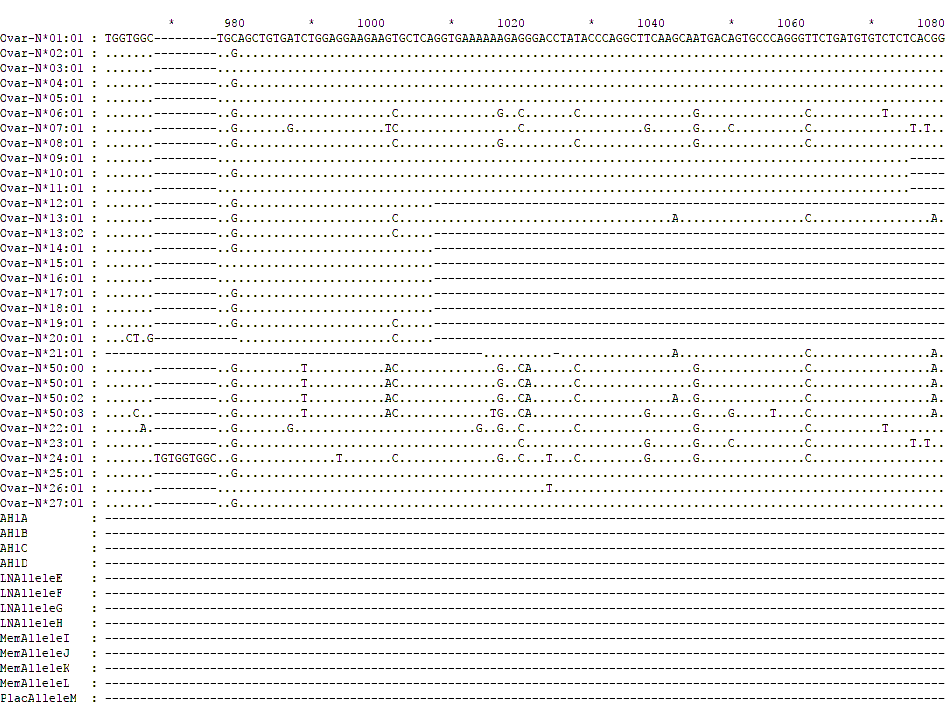


**Supplementary Figure 3 Pair-wise alignment of the current MHC class I amino acid sequences.**

Pairwise alignment of the predicted amino acid sequences from each of the MHC class I transcripts identified in this study along with those archived in the IPD-MHC Database. Classical MHC class I alleles are named Ovar-N*01 to Ovar-N*27 while the non-classical alleles are named Ovar-N*50. The amino acid sequence predicted from the *Ovar-N*01:01* transcript has been used as the consensus sequence. Gaps and missing data are represented by a hyphen (-) and identity to the reference sequence is represented by a period (.) Sequences isolated from the AH-1 trophoblast cell line are shown with grey shading. Full length transcripts obtained are listed with their official nomenclature while the internal fragments are names in alphabetical order depending on their tissue of origin. Sequences derived from lymph node, inter-cotyledonary membrane and placentome are defined as LN, Mem and Plac respectively.

**
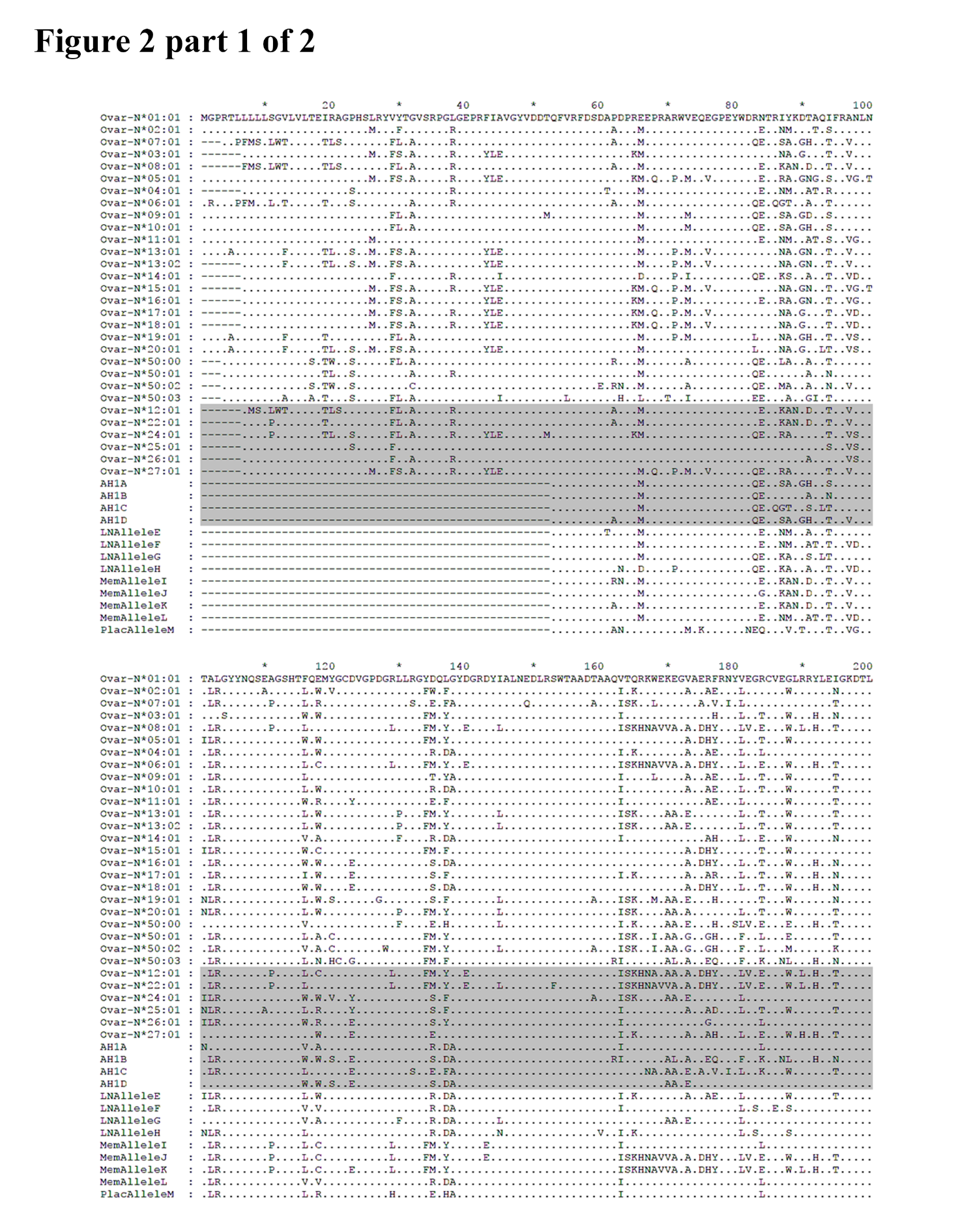
**

**
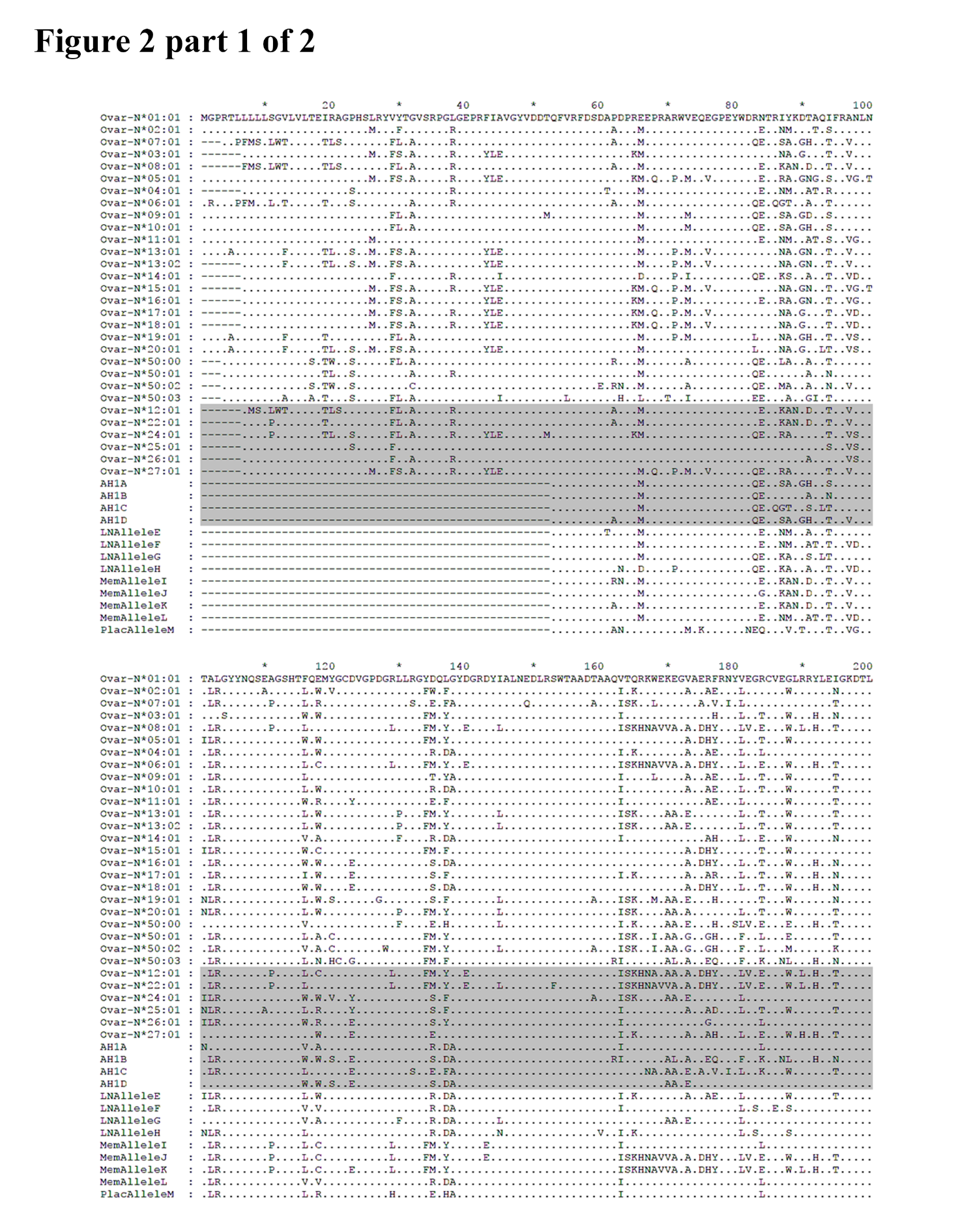
**

**
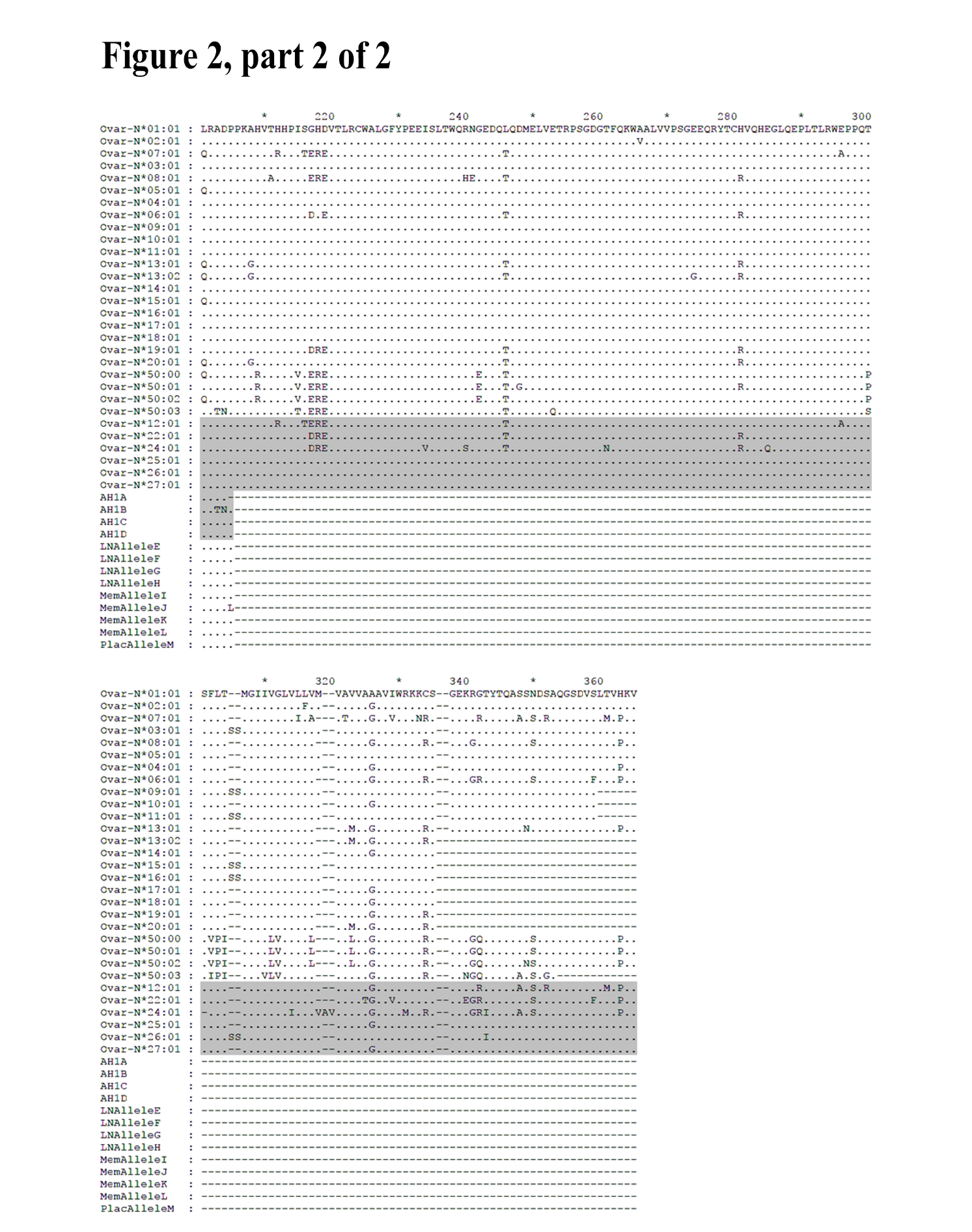
**

**
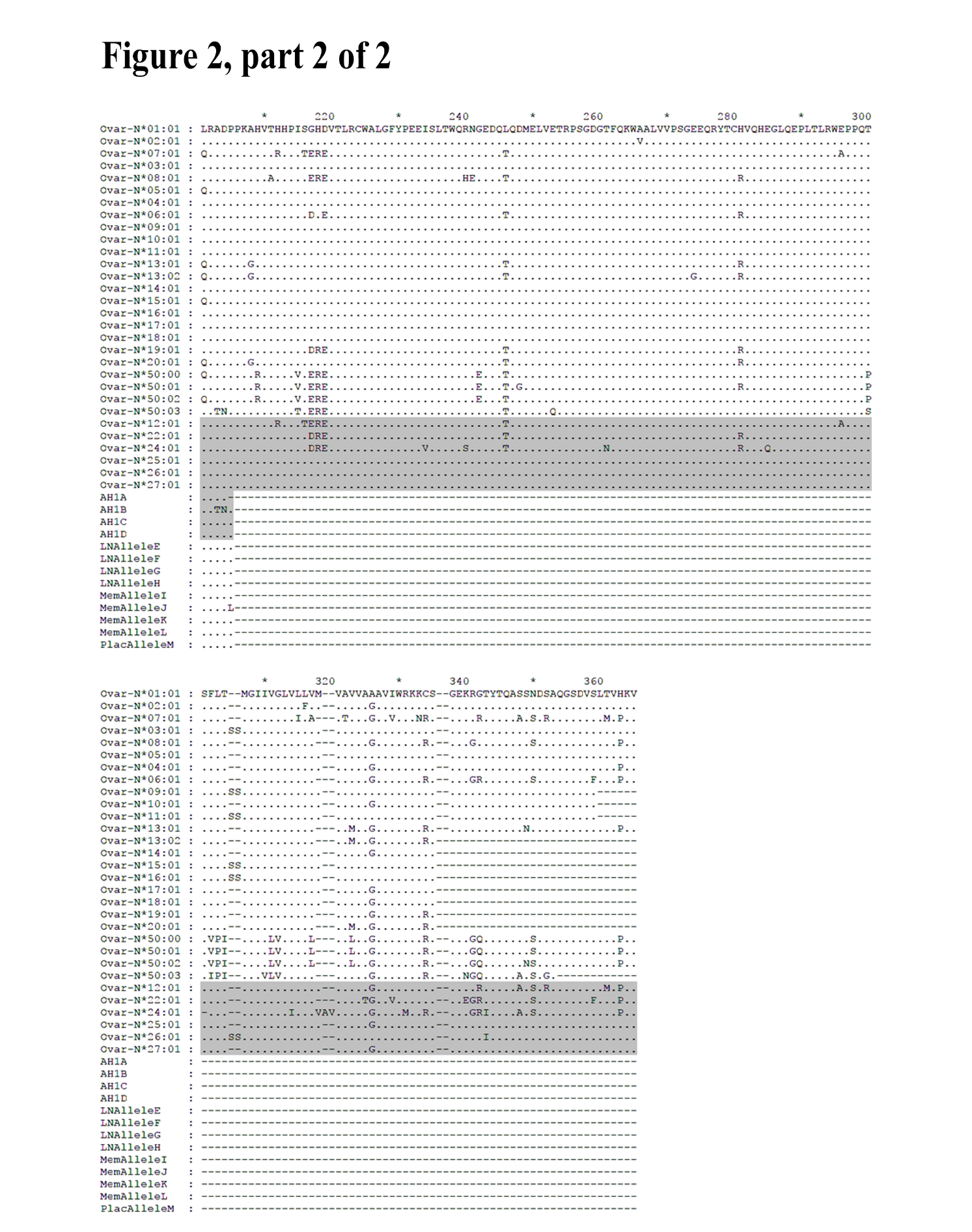
**

**Supplementary Figure 4 Serial section of mesenteric lymph node stained with isotype-control mAb to serve as the immunohistochemistry negative control for NKp46/NCR1 staining.**

Frozen sections of the medial iliac lymph node collected at post-mortem from two ewes infected with *Nematodirus battus* to serve as control tissues. (described in detail in section Animals and tissues). Serial sections were labelled with the Border Disease Virus mAb (isotype-matched control to the ovine NKp46/NCR1 mAb labelling displayed in Figure4). There is no brown labelling in the paracorex area surrounding the lymphoid follicle (asterisk).

**
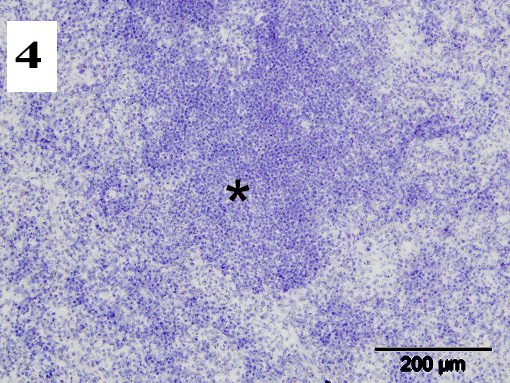
Supplementary Table 1 Summary of NKp46/NCR1+ve cell expression in ovine placental tissues and lymph nodes.**

Determination of NKp46/NCR1+ve staining for the ovine lymph node and placental tissues (inter-cotyledonary membrane and placentome tissue samples) when compared directly to the mesenteric lymph node tissues of *Nematodirus battus*-infected ewes stained with NKp46/NCR1 mAb (Figure 4A) or Border Disease Virus specific mAb (isotype-matched control mAb, Supplementary Figure 4). This table summarises the data from the seven ewes. For the uterine wall tissue, up to two separate sections from each animal were tested and the results presented in separate rows of table. For the inter-cotyledonary membrane and placentome tissues, one tissue section was taken from each placenta. There were up to two placentas for each animal tested, and the results are presented in separate rows of the table.

|  | Animal | | | | | | | |
| --- | --- | --- | --- | --- | --- | --- | --- | --- |
| Tissue | 1 | 2 | 3 | 4 | 5 | 6 | 7 | Staining |
| Right-prefemoral lymph node | + | + | No sample | + | + | + | + | Positive |
| Lumbo-aortic lymph node | + | + | No sample | + | + | + | + | Positive |
| Medial iliac lymph node | + | - | No sample | - | + | - | + | Varied positive/ negative |
| Uterine wall | No  Sample | - | - | - | - | - | - | Negative |
|  | No  sample | No  sample | - | - | - | - | - |  |
| Inter-cotyledonary membrane | No sample | - | - | - | - | - | - | Negative |
|  | No sample | No Sample | - | - | - | - | - |  |
| Placentome | No sample | - | - | - | - | - | - | Negative |
|  | No sample | No sample | - | - | - | - | - |  |

**
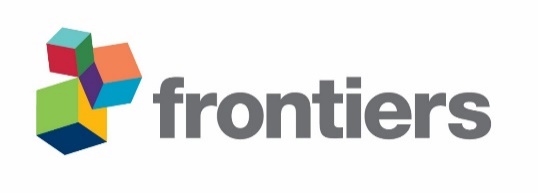
**
